# Mucormycosis in a Renal Transplant Recipient: Case Report and Comprehensive Review of Literature

**DOI:** 10.1155/2014/950643

**Published:** 2014-02-12

**Authors:** Tamim Hamdi, Vanji Karthikeyan, George J. Alangaden

**Affiliations:** ^1^Division of Nephrology and Hypertension, Department of Internal Medicine, Henry Ford Hospital, 2799 West Grand Boulevard, CFP-514, Detroit, MI 48202, USA; ^2^Division of Infectious Diseases, Department of Internal Medicine, Henry Ford Hospital, Detroit, MI 48202, USA

## Abstract

Mucormycosis is a rare but devastating infection. We present a case of fatal disseminated mucormycosis infection in a renal transplant patient. Uncontrolled diabetes mellitus and immunosuppression are the major predisposing factors to infection with Mucorales. Mucorales are angioinvasive and can infect any organ system. Lungs are the predominant site of infection in solid organ transplant recipients. Prompt diagnosis is challenging and influences outcome. Treatment involves a combination of surgical and medical therapies. Amphotericin B remains the cornerstone in the medical management of mucormycosis, although other agents have been used. Newer agents are promising.

## 1. Introduction

Invasive fungal infections (IFIs) occur in up to 20% of recipients of renal transplantation [1] and remain a diagnostic and therapeutic challenge. While *Candida* infections are the most common [2], invasive aspergillosis is the most fatal, with a mortality rate reaching 75% [3]. According to the TRANSNET report, a recent prospective and comprehensive study of invasive non-*Aspergillus* fungal infections, mucormycosis is much less common, occurring in 3 of 8494 renal transplants between 2001 and 2006 and accounting for 28 of 1208 cases of IFIs among all solid organ transplant (SOT) recipients [4]. Fungal infections in general occur in the intermediate (1–6 months) to late (more than 6 months) posttransplant period [5]; 37.8% of non-*Aspergillus* infections were reported to occur within the first 6 months and 33.3% two years after the transplant [4]. A recent prospective study reported a median time to infection with mucormycosis of 5.7 months in nonliver SOT recipients [6].

We report a case of a renal transplant recipient who developed rapidly progressive and fatal disseminated mucormycosis one month posttransplant.

## 2. A Case Report

A 48-year-old African-American male presented with progressive generalized weakness 3 months after receiving a living unrelated kidney transplant for end-stage kidney disease of unclear etiology. He was maintained on hemodialysis for 4 years prior to the transplant.

The patient received induction immunosuppression (IS) with antithymocyte globulin and methylprednisolone dosed per our institution's protocol. Maintenance IS consisted of mycophenolate mofetil, tacrolimus, and corticosteroids, in addition to trimethoprim/sulfamethoxazole and valganciclovir for microbial prophylaxis. The transplant was complicated by delayed graft function requiring hemodialysis. On postoperative day 7, revision of the ureterocystostomy and insertion of a double J-ureteral stent was performed for ureteral obstruction. A kidney biopsy showed no evidence of acute rejection. On the day of discharge, the serum creatinine reached a nadir of 159.12 µmol/L. Iron studies on the same day showed a serum ferritin of 1815 ng/mL with an iron saturation of 63%. The ureteral stent was removed 20 days after placement. No relevant anomalies were detected on multiple chest radiographies. During followup, he developed new-onset diabetes mellitus and his serum bicarbonate was noted to be within normal range.

**Figure 1:**
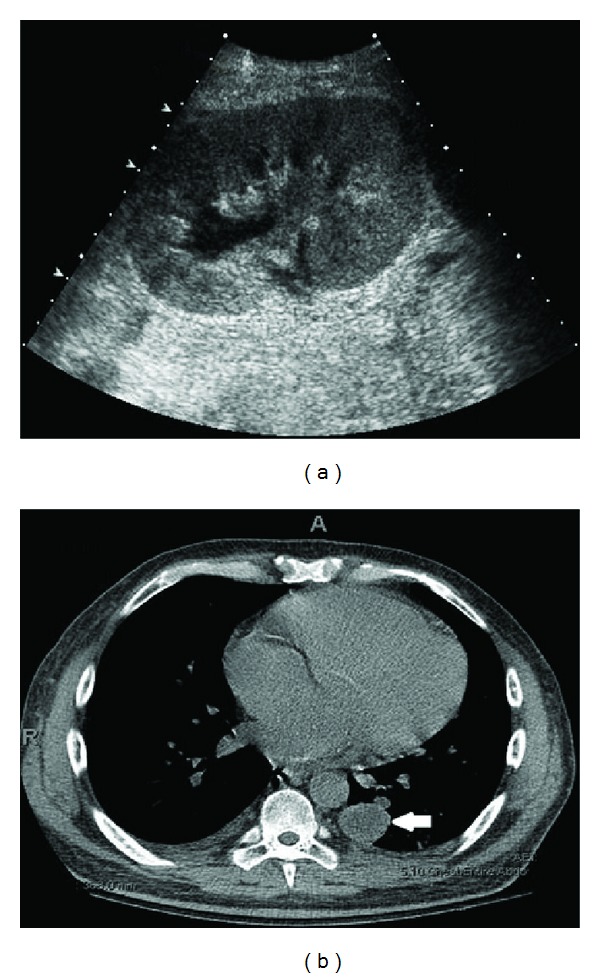
(a) Ultrasound showing enlargement of the transplanted kidney; (b) CT scan of the chest showing a cystic mass in the lower lobe of the left lung (white arrow).

**Figure 2:**
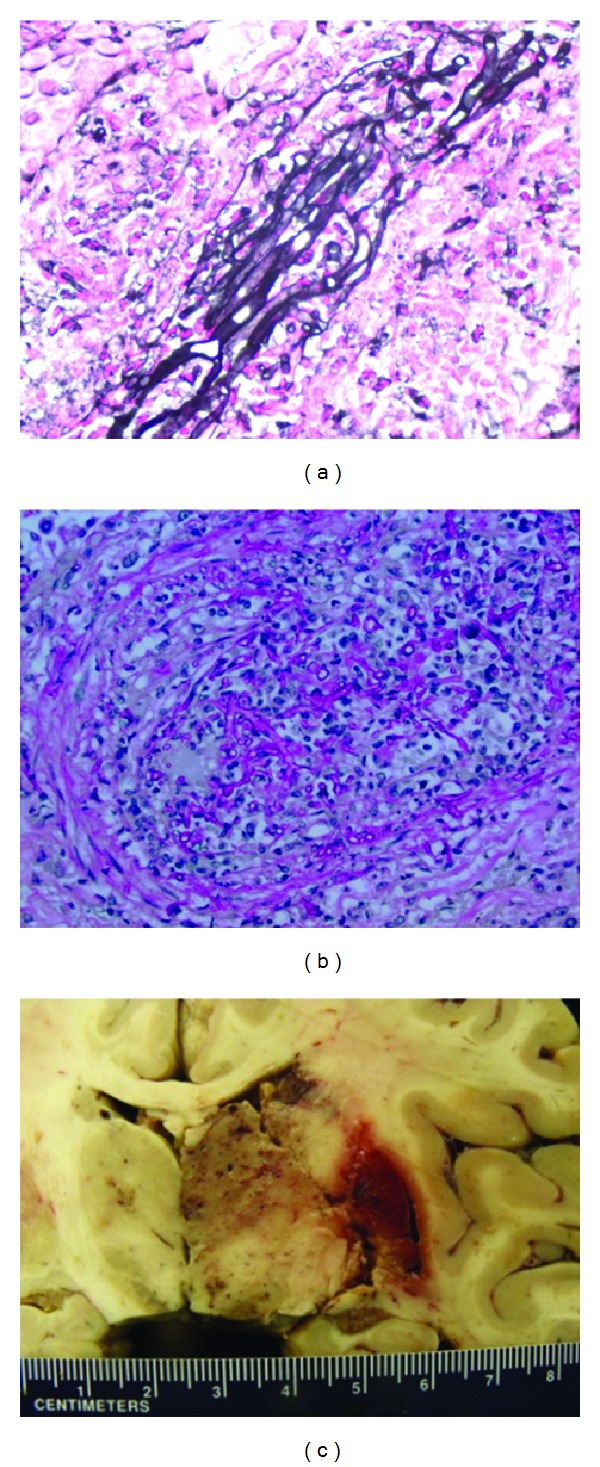
Light microscopy of the transplanted kidney biopsy ((a), Jones stain, 400x) and lung tissue ((b), PAS stain, 400x) showing fungal hyphae. (c) Necrotic and hemorrhagic lesion in the right cerebral hemisphere.

At the time of the current admission, the patient suffered acute kidney injury with serum creatinine of 406.64 µmol/L. There was no evidence of volume depletion or tacrolimus toxicity. On admission his blood glucose level was 24.29 mmol/L and required three days to be adequately controlled. Renal ultrasound showed an enlarged transplanted kidney (Figure 1). A renal biopsy demonstrated acute fungal pyelonephritis, with severe destructive acute granulomatous inflammation involving all of the renal elements. Numerous branching nonseptate fungal hyphae were seen throughout the renal tissue (Figure 2). The fungal morphology was suggestive of Mucorales. Computed tomography (CT) of the chest showed bilateral ground-glass infiltrates, multiple nodular opacities, and a 3.1 × 3 cm cystic mass in the lower lobe of the left lung (Figure 1). CT of the head showed no involvement of the sino-orbital areas or the brain. The patient was started on liposomal amphotericin B (5 mg/kg daily) for suspected disseminated mucormycosis. Mycophenolate and tacrolimus were discontinued and the dose of prednisone reduced. Due to persistent neutropenia (absolute neutrophil count <500 cell/mm^3^), trimethoprim/sulfamethoxazole and valganciclovir were discontinued and the patient was started on filgrastim. A nephrostomy tube was inserted because of persistent hydronephrosis. Urine cultures obtained from both the nephrostomy and the urinary catheter did not demonstrate any microbial growth. The patient's renal function worsened rapidly and amphotericin B was discontinued. He was enrolled in an open-label phase III clinical study of isavuconazole, a novel nonnephrotoxic broad-spectrum anti-fungal azole, with good activity against mucorales. On the fourth hospital day, the patient became progressively dyspneic and tachycardic necessitating transfer to the intensive care unit where he required mechanical ventilation. Cultures of the bronchoalveolar lavage (BAL) fluid were negative. Serum and BAL *Aspergillus* galactomannan antigen assays were negative. Polymerase chain reaction (PCR) assay of the serum was negative for cytomegalovirus DNA. Despite maximal vasopressor and mechanical ventilatory support, the patient died on day 6 of his hospital stay.

Autopsy showed multiple renal cortical hemorrhagic lesions with black discoloration of the papillae and multiple hemorrhagic lesions in the lungs with abscess cavity in the left lower lobe. Brain involvement was noted by the presence of a 6 × 5 × 5 cm hemorrhagic and necrotic lesion in the right cerebral hemisphere with obliteration of blood vessels by matted fungal hyphae (Figure 2). *Mycocladus corymbifer* (formerly *Absidia corymbifera*) was isolated on tissue cultures from the kidney biopsy and the lung tissue obtained by autopsy (Figure 2). The patient had no recent history of travel or outdoor activities including camping, hiking, and visits to caves, lakes, or river banks. His wife noted that she recently cleaned the house windows of molds.

## 3. Discussion

### 3.1. Epidemiology and Risk Factors

Mucorales are ubiquitous in nature and rarely cause disease in immunocompetent hosts, except in the settings of uncontrolled diabetes mellitus [7], heavy exposure as in natural disasters, [8, 9] or rarely without apparent predisposing factors [7, 10, 11]. Recipients of SOT are at higher risk given their multiple predisposing factors. Diabetes mellitus remains the leading risk factor among all studied patient populations, as 36% of Roden et al.'s 929 cases were diabetic, mostly type 2, and in the setting of ketoacidosis [7]. Even among the SOT and hematopoietic stem cell transplant (HSCT) recipients developin gmucormycosis, the prevalence of diabetes mellitus was 43.8%. In a recent prospective study, the odds ratio (OR) for developing mucormycosis among diabetics with SOT was 8.11, compared to nondiabetic matched SOT recipients [6]. Thus it remains an independent risk factor even in the presence of other predisposing factors [12]. Another major risk factor is the state of immunosuppression, especially the use of potent T cell depleting agents [13, 14] and the presence of neutropenia [1]. In the TRANSNET report, 50.6% of SOT and HSCT patients developing mucormycosis were neutropenic within 60 days prior to the onset of infection. Initial or subsequent graft rejection necessitating augmented IS, a condition commonly encountered in renal transplant recipients, was also associated with an increased risk of mucormycosis [4, 15–17]. Renal failure and prior exposure to caspofungin or voriconazole (antifungal agents with no activity against Mucorales) increase the risk of mucormycosis (OR 3.17 and 4.41, resp.) [6], as well as the use of ureteral stents during renal transplant and prolonged ICU stay [1]. Use of tacrolimus was associated with a 4-fold reduction in the risk of developing mucormycosis [6]. This could be explained by the synergy demonstrated in vitro between calcineurin inhibitors and triazole antifungals against some species of mucormycosis [18, 19]. Our patient had numerous risk factors associated with mucormycosis including new-onset poorly controlled diabetes after transplantation, induction with T-cell depleting agent, severe neutropenia, abnormal renal function, ICU stay, and ureteral stenting.

### 3.2. Clinical Manifestations

In the reported literature, the species of Mucorales accounting for most of the cases is variable, likely reflecting regional and hospital variability. *Rhizopus* species is the most common, accounting for 35%–73% of cases, followed by *Mucor* (13%–37%) and *Mycocladus* (0%–13%) [4, 6, 7, 15]. The infection is acquired through inhalation of spores or rarely through direct contact with the skin. The hyphae of pathogenic Mucorales are angioinvasive, which lead to hemorrhagic necrosis, vascular thrombosis, and tissue infarction [1, 9]. The primary site of infection varies according to the host's condition. Localized sinonasal or sino-orbital disease with involvement of the brain accounts for 66% of mucormycois in diabetic patients. However, pulmonary infection is the predominant site affected in recipients of SOT [4, 7], accounting for 39% of cases with involvement of other organ sites in 48% [6]. Mucormycosis can virtually involve every organ, such as the skin, gastrointestinal (GI), cardiovascular, genitourinary, and musculoskeletal systems as well as infections of surgical wounds and intravascular catheter exit sites [1, 4, 7, 20, 21]. Of note, isolated renal infection without systemic involvement has been attributed to seeding during transient periods of fungemia [7, 22–24]. Donor-derived infection through transmission with the allograft is also possible [25, 26], but it presents early after transplantation [27].

Dissemination, defined as infection at two or more non-contiguous sites [7, 28], is a catastrophic complication that carries a grave prognosis. The risk of dissemination is directly linked to the primary site of infection and the type of transplant [6]. In the SOT population, disseminated disease occurs in 9–26% of cases, with the highest incidence among liver transplant recipients (26–55%), followed by lung (11–25%), heart (11–20%), and kidney transplant recipients (9–13%) [6, 7, 12, 15]. The unique factors that increase the risk of dissemination in specific organ transplant groups remain undefined. The risk of dissemination based on the primary site of infection among SOT recipients is unknown but has been reported among all cases of mucormycosis, including but not exclusive to SOT [7]. About 50% of patients with pulmonary infection, 38% with GI infection, and 20% with cutaneous infections suffered from dissemination [7].

### 3.3. Diagnosis

The diagnosis of mucormycosis is challenging and often delayed [29, 36], as the clinical presentation is not specific and symptoms and signs are often muted by the blunted immune response. Timely diagnosis and treatment are crucial due to the aggressive course of mucormycosis that may eventually lead to tissue necrosis and dissemination. The clinical signs and symptoms are related to the site of disease. Pulmonary disease can present with fever, pleuritic chest pain, or features of pneumonia. Rhino-sino-orbital disease presents with facial or orbital pain and swelling, proptosis, visual loss, and ophthalmoplegias. Given the angioinvasive nature of Mucorales infection, rapidly progressive necrotic lesions caused by infarction of the tissue can be seen in the nasal and sinus mucosa. Involvement of the brain can cause features suggestive of stroke, cranial nerve palsies, altered mentation, headaches, and seizures. A high index of suspicion and suggestive signs and symptoms are needed, and the diagnosis is confirmed by a combination of radiological, histological, and microbiological studies. Plain or contrast-enhanced CT or magnetic resonance imaging (MRI) of the head, sinuses, brain, chest, and abdomen may show some suggestive radiological signs [1, 13]. CT features of pulmonary mucormycosis in SOT recipients commonly include consolidation or mass-like lesions, nodules, or cavities in about 25% of patients [37]. Opacification of the sinuses is seen in sinonasal disease with involvement of the maxillary sinuses being the most common, followed by the ethmoid and sphenoid sinuses [38]. Cerebral disease generally involves the frontal lobes [38]. Tissue biopsy is needed to confirm the etiological diagnosis, and direct identification of the organism by culture or histopathology is the gold standard. The hyphae of Mucorales are broad, irregularly branched, thin-walled, and sparsely septate (Figure 1). The latter might explain the fragility of the hyphae and the low sensitivity of cultures, reported to be 50% in earlier studies [7]. However, the improvement in laboratory techniques has increased the yield of cultures, recently reported to reach 92% [6]. Blood cultures are generally negative. Molecular diagnostic tests for identification of Mucorales are increasingly used for early detection of infection and identification of genus even in cases when cultures are negative. Qualitative and quantitative PCR has been used to detect infection in lung tissue, bronchoalveolar lavage fluid, and serum samples [39]. Whether these techniques can be recommended for routine use remains unclear, although PCR testing should be considered in cases where the histopathology is suggestive of Mucorales, but cultures are negative [39–41]. Mucorales do not release *β*-D-glucan during their growth, so a positive galactomannan serum assay indicates coinfection with *Aspergillus* [42].

### 3.4. Treatment

Timely initiation of treatment is crucial and associated with better survival [36]. The optimal management of mucormycosis is based upon early recognition and initiation of treatment, surgical resection of necrotic tissue if possible, and reversal of predisposing factors, such as uncontrolled glycemia, IS, and neutropenia.

Surgery is an essential part of the management of localized disease, such as rhino-orbito-cerebral disease, and surgical resection and debridement are associated with improved outcomes [7]. Lobectomy and nephrectomy were reported to be successful in isolated pulmonary [29, 43] and renal disease, respectively [22]. Salvage hepatic resection and re-transplantation have also been reported [44].

Amphotericin B (AmB) and posaconazole are the only antifungal agents currently available that are active against Mucorales. AmB is considered the drug of choice. Lipid formulations of AmB are thought to have better activity and safety profile compared to conventional AmB deoxycholate in murine models and patients with hematologic malignancies [31, 45]. Treatment with AmB lipid complex (ABLC) was reported to be successful in 8 of 14 (57%) SOT recipients with mucormycosis [6, 29], compared to 16 of 17 patients (94%) treated with liposomal AmB (LAmB) [6]. In a recent retrospective series of 41 patients with rhino-orbital mucormycosis with and without cerebral involvement (including 2 renal and 2 HSC transplants), ABLC was successful in only 7 of 22 patients (32%), compared to 13 of 19 patients (68%) treated with AmB deoxycholate or LAmB [30]. The discrepancy was attributed to poor central nervous system penetration of ABLC [46]. In the same study, the rate of nephrotoxicity (56%) was similar among patients treated with various formulations of AmB. This might be explained by the use of LAmB and ABLC in doses up to 10 mg/kg in some patients [30]. The superiority of LAmB was also observed in a retrospective series of 59 patients with hematological malignancies and mucormycosis, where treatment with LAmB was successful in 58% of patients compared to 23% for AmB deoxycholate [31]. The reduced nephrotoxicity associated with standard doses of LAmB would make it the primary agent for treatment in SOT recipients, as these patients are usually receiving other potentially nephrotoxic agents such as calcineurin inhibitors. LAmB would also be preferred in renal transplant recipients where nephrotoxicity can result in graft failure.  Table 1 summarizes the success rate among different patient populations treated with various antifungal regimens.

Posaconazole is available in oral formulation only and has been used as an oral step-down agent after successful response with AmB or for salvage therapy in case of refractory disease or intolerance to side effects of AmB [32, 33]. In a retrospective study, 91 patients with mucormycosis (including 10 SOTs) received at least 30 days of enteric posaconazole at 400 mg twice a day as a salvage therapy. At 12 weeks, total response rate was 60%, of which complete and partial responses accounted for 14% and 46%, respectively. The disease remained stable in 21% of patients and progressed in 17%, and the outcome in the remaining 2% was not known [32]. Similarly, Greenberg et al. [33] reported a success rate of 79% (19/24), which decreased to 25% in those with disseminated disease. In light of these findings, Peel et al. [34] successfully used posaconazole as a first-line agent on a patient with systemic lupus erythematosus and mucormycosis, as did Singh et al. [6] on 3 of 5 patients (60%). The initiation of posaconazole as first-line therapy for the treatment of serious fungal infections remains problematic. Posaconazole is presently available for oral administration only. The oral bioavailability is enhanced when administered with a fully fatty meal and with a lower stomach acid pH [47]. Hence posaconazole has to be administered soon after a full meal especially with fatty foods, liquid nutritional supplements, or an acidic carbonated beverage [48]. Therapeutic drug monitoring may be important in optimizing outcomes due to erratic absorption resulting in unpredictable levels. This is especially true in the presence of a concentration-effect relationship [47]. The dietary requirements for optimal absorption and achievement of therapeutic drug levels can be difficult in transplant recipients receiving several other oral medications.

Various outcomes have been reported using different combination therapies with a small number of patients. Combination of LAmB with posaconazole was no more effective than LAmB alone in a murine model of mucormycosis [49]. Singh et al. [6] reported that the treatment success rate was significantly lower with combination therapy compared to LAmB alone. However, most of the patients in the combination group had disseminated disease and were therefore more ill at baseline.

Although echinocandins are reported to have no to moderate activity against Mucorales [30, 50–54], the use of an echinocandin in combination therapy has been attempted. Combined with a median of 2 surgical procedures per patient, Reed et al. [30] reported a 100% success rate (6/6) with the combination of caspofungin with a lipid AmB, compared to 45% (14/31) success rate for those treated with ABLC monotherapy. The patients with cerebral involvement witnessed the most benefit, with a survival rate of 100% (4/4) in the combination group, versus 25% (4/16) in the monotherapy group. In a recent expert review [41], the combination of an echinocandin with LAmB was recommended as an initial induction therapy for 3 weeks, followed by a step-down period with oral posaconazole [41].

The role of granulocyte colony-stimulating factor (G-CSF) in the reversal of neutropenia and recovery from mucormycosis is not clear. Small reports described a parallel improvement in clinical manifestations and neutrophil count. However, neutropenia was not associated with treatment failure by univariate analysis [6], and reversal of neutropenia was not associated with treatment success by multivariate analysis [31].

Isavuconazole is a new broad-spectrum triazole antifungal agent that has good *in vitro* activity against clinically important yeasts and molds including *Aspergillus* and Mucorales. The drug has a favorable pharmacokinetic profile, is available as an intravenous and oral formulation, and has the advantage of less drug-drug interaction than voriconazole and posaconazole. It is presently in phase III clinical trials for the treatment of invasive aspergillosis and other molds [55]. Our patient was enrolled in a phase III open-label clinical trial of isavuconazole for the treatment of mold infection as he developed rapidly progressive nephrotoxicity with LAmB therapy.

Mucormycosis occurs in patients with iron overload, as host iron availability is important for the pathogenesis of mucormycosis. Adjunctive therapy with deferasirox iron chelation and LAmB had shown improved outcomes in the diabetic mouse model of mucormycosis. However, a small clinical trial of 20 patients (DEFEAT Mucor Study) using LAmB and deferasirox demonstrated worse outcomes in the deferasirox arm [56].

Unlike recipients of liver or lung transplants, especially vulnerable to invasive *Candida* and *Aspergillus*, the risk of IFI in kidney transplants recipients is low. Currently, there are no recommendations regarding routine prophylaxis against fungal infections in kidney transplant recipients.

### 3.5. Prognosis

The overall mortality rate of mucormycosis ranges from 38% to 56.5%. The primary site of infection plays a major role in determining the outcome, with marked increase in mortality when dissemination occurs, reportedly up to 100%. Mortality has been reported from 33% to 60% for isolated pulmonary infection, but 95% when disseminated, 85% to 100% for GI infection, 10% to 17% for cutaneous infection (94% when disseminated), and 31% to 93.3% for rhinocerebral infection (98% when disseminated to the central nervous system) [6, 7, 15]. In a series of six cases of renal transplant and mucormycosis, all patients lost their graft function, including the three who survived. This was attributed to reduction in IS and amphotericin B-induced nephrotoxicity [29]. The species of mucormycosis also affect outcome, with the highest treatment success rate achieved with *Rhizopus* species, followed by *Mucor* and *Mycocladus* (68%, 59%, and 50%, resp.) [6].  Table 2 summarizes factors reported to affect outcome. Interestingly, in a review of literature by Almyroudis et al. [15], the mortality rate in patients on maintenance IS was equivalent to that in patients who received induction IS or treatment for acute rejection within 1 month prior to diagnosis, respectively (28 of 65 (43%) versus 29 of 50 (58%); NS). They also reported that the discontinuation or reduction of IS was associated with a better survival rate (32 of 46 (69.5%) versus 12 of 26 (46.1%); *P* = 0.05). This reflects that the state of IS may carry the same risk of infection even long after induction and may explain why 33.3% of non-*Aspergillus* infections in the TRANSNET study occurred more than 2 years after transplant [4]. Despite the timely diagnosis in the case presented, we were not able to save our patient. The combination of multiple risk factors and the presence of dissemination likely since the time of presentation eventually lead to the grave outcome.

## 4. Conclusion

Mucormycosis is a rare but serious infection in renal transplant recipients as illustrated in our case report. Uncontrolled diabetes mellitus and IS are the most common risk factors. Diagnosis of mucormycosis is based on a high index of suspicion, along with a combination of appropriate imaging, histopathologic identification, microbiology, and newer molecular diagnostic tests. Surgical debridement is important in localized disease. Therapy with liposomal AmB remains the cornerstone of treatment, with posaconazole used as a second-line option in case of treatment failure or intolerance. The role of combination therapy and newer agents such as isavuconazole therapy remains to be defined. Despite early diagnosis and treatment, graft loss and mortality rate remain high in patients with disseminated disease.

## Figures and Tables

**Table 1:** Success rate of antifungal agents used to treat mucormycosis.

Treatment	Study	Number of patients and patient population	Dosage	Success rate
ABLC	Singh et al. [6]^a^	50, SOTs	NR	5/8 (62%)
	Forrest and Mankes [29]	6, KTs	5–10 mg/kg	3/6 (50%)
	Reed et al. [30]	41, ROM/ROCM (2 KTs, 2 HSCTs)	5–10 mg/kg	7/22 (32%)

LAmB	Singh et al. [6]	50, SOTs	NR	16/17 (94%)
	Reed et al. [30]^b^	41, ROM/ROCM (2 KTs, 2 HSCTs)	5–10 mg/kg	13/19 (68%)
	Pagano et al. [31]	59, hematologic malignancy	3 mg/kg	7/12 (58%)

AmB deoxycholate	Singh et al. [6]	50, SOTs	NR	3/5 (60%)
	Reed et al. [30]^b^	41, ROM/ROCM (2 KTs, 2 HSCTs)	1 mg/kg	13/19 (68%)
	Pagano et al. [31]	59, hematologic malignancy	3 mg/kg	9/39 (23%)

Posaconazole monotherapy as second line	Van Burik et al. [32]	91, (10 SOTs)	800 mg daily	CR: 13/91 (14%) PR: 42/91 (46%)
	Greenberg et al. [33]	24, (4 SOTs)	800 mg daily	19/24 (79%)

Posaconazole monotherapy as first line	Peel et el. [34]	1, patient with SLE	800 mg daily	1/1 (100%)
	Singh et al. [6]	5, SOTs	NR	3/5 (60%)

LAmB and posaconazole	Singh et al. [6]	5, SOTs	NR	2/5 (40%)
	Rickerts et al. [35]	1, AML	5 mg/kg and 800 mg daily	1/1 (100%)

ABLC and caspofungin	Reed et al. [30]	41, ROM/ROCM (2 KTs, 2 HSCTs)	5 mg/kg; NR	6/6 (100%) versus 14/31 (45%) for ABLC alone

ABLC: amphotericin B lipid complex; LAmB: liposomal AmB; AmB: amphotericin B; NR: not reported. ROCM: rhino-orbito-cerebral mucormycosis; SOT: solid organ transplant; KT: kidney transplant; HSCT: hematopoietic stem cell transplant. ^a^The only prospective study; ^ b^success rate was combined for both treatment groups. CR: complete response; PR: partial response; AML: acute myeloid leukemia.

**Table 2:** Factors affecting outcome of Mucormycosis infection.

Treatment failure	Treatment success
Dissemination (OR = 11.21 [7], 14.6 [6])	Use of liposomal AmB (OR = 0.23, [6] RR = 0.5 [31])
Renal failure (OR = 11.3 [6], 7.16 [7])	Surgical resection (OR = 0.03 [6])
	Combination of AmB and surgery [15]
	Discontinuation or reduction of IS [15]

OR: odds ratio; AmB: liposomal amphotericin B; RR: relative risk; IS: immunosuppression.
